# Globally invariant behavior of oncogenes and random genes at population but not at single cell level

**DOI:** 10.1038/s41540-023-00290-9

**Published:** 2023-06-24

**Authors:** Olga Sirbu, Mohamed Helmy, Alessandro Giuliani, Kumar Selvarajoo

**Affiliations:** 1 Bioinformatics Institute (BII), Agency for Science, Technology and Research (A*STAR), Singapore, 138671 Republic of Singapore; 2 Department of Computer Science, Lakehead University, Thunder Bay, ON P7B 5E1 Canada; 3 Environment and Health Department, Istituto Superiore di Sanità, Viale Regina Elena 299, 00161 Roma, Italy; 4 Synthetic Biology for Clinical and Technological Innovation (SynCTI), National University of Singapore (NUS), Singapore, 117456 Republic of Singapore; 5 School of Biological Sciences, Nanyang Technological University (NTU), Singapore, 639798 Republic of Singapore

**Keywords:** Cancer, Computational biology and bioinformatics

## Abstract

Cancer is widely considered a genetic disease. Notably, recent works have highlighted that every human gene may possibly be associated with cancer. Thus, the distinction between genes that drive oncogenesis and those that are associated to the disease, but do not play a role, requires attention. Here we investigated single cells and bulk (cell-population) datasets of several cancer transcriptomes and proteomes in relation to their healthy counterparts. When analyzed by machine learning and statistical approaches in bulk datasets, both general and cancer-specific oncogenes, as defined by the Cancer Genes Census, show invariant behavior to randomly selected gene sets of the same size for all cancers. However, when protein–protein interaction analyses were performed, the oncogenes-derived networks show higher connectivity than those relative to random genes. Moreover, at single-cell scale, we observe variant behavior in a subset of oncogenes for each considered cancer type. Moving forward, we concur that the role of oncogenes needs to be further scrutinized by adopting protein causality and higher-resolution single-cell analyses.

## Introduction

Cancer is both a heterogeneous as well as a highly dynamic malady^1–4^. Even within the same cancer types, tumors can exhibit *intratumoral* variations^5^ (biological variations gained during the progression of the disease, i.e., variable histopathology), *intertumoral* variations^6^ (variation within the same cancer patient i.e., metastasis), and *interpatient* variations^7^ (variations among patients i.e., heterogeneity). Furthermore, the lack of a clear understanding of cancer causality is another confounding factor that makes cancer research complex^8^. Due to this, and despite tremendous efforts, approaching the study and treatment of cancer is a delicate ordeal, with existing treatments not being foolproof and often working sporadically only on a subset of patients^3^.

To address these challenges, researchers target genes that are associated with the hallmarks of cancer, such as increased cell proliferation and avoidance of cell death^9^. Proto-oncogenes represent an exemplar case as they are genes whose expression is fully physiological and only when mutated (with a consequent dysregulation of their original function) act as cancer-causing elements^10^. This is the case of tumor suppressor genes (TSG) whose original function of limiting cell proliferation and directing cells toward apoptosis, when abolished by mutation, promote cancer development^11^.

The high interest in cancer research also adds complexity which is evident by the lack of a unified consensus definition of oncogenes. For example, the National Human Genome Research Institute’s definition is, “an oncogene is a mutated gene that has the potential to cause cancer”^12^, while the National Cancer Institute (NCI) defines them as, “a gene that is a mutated (changed) form of a gene involved in normal cell growth”^11^. NCI provides another broader definition, “an oncogene is a gene that has the potential to cause cancer without the requirement of a particular change in the gene sequence or in the gene expression”. Lastly, in *Comprehensive Toxicology* the term oncogene refers to “a gene that encodes a protein that is capable of transforming cells in cultures or inducing cancer in animals”^13^.

For cataloging oncogenes, the Cancer Genes Census (CGC) lists genes that contain mutations “that have been causally implicated in cancer”^14^. According to the CGC, less than 1% of the human protein-coding genes, 729 genes, are implicated in cancer, and they are divided into two tiers based on the availability of documented activity and curated evidence of “promoting oncogenic transformation”. The Atlas of Genetics and Cytogenetics in Oncology and Hematology (AGCOH) gives more than double estimates of oncogenes number, providing a list of 1580 genes, annotated as cancer genes, and a larger list of over 27 K genes that are “possibly implicated in cancer”^15^. Thus, it becomes apparent that the way oncogenes are defined impacts the inclusion and exclusion of genes in these databases, drawing attention to them in research and therapeutics, the problem being further exacerbated by the exclusion of non-coding genes that have been more recently associated with cancer^16^.

The current challenges associated with navigating the massive body of research become even more evident in a recent study that found 87.7% of human genes have been associated with cancer to some extent^17^. This is not surprising given the prevalence of cancer research with a yearly estimate of 200,000 cancer-related publications^17^, the complexity, and heterogeneity associated with cancer, as well as the flexible definition of oncogenes. However, not only it is unlikely that all these genes can be classified as cancer genes, but the large number and lack of consensus makes the road to finding a cure even more challenging, while clinically relevant goals, such as disease prediction and prevention, become more difficult to achieve^18^.

Here, we focus solely on the curated list of oncogenes from the CGC database^14^ to which we referred to as CGC genes, and cancer-specific oncogenes (CSO) which we identified for each cancer type from the CGC list using the identifiers provided by the database. In this paper, we, thus, investigated the relationship between cancer and cancer-associated genes, to find if the latter behave any differently from the rest of the genes when we compared normal and cancer proteomic and transcriptomic data. This comparison encompassed multiple data analytics (correlation, noise estimation, dimensionality reduction etc.), sample similarity analysis, protein–protein interaction network analysis on bulk as well as single-cell RNA-seq gene expression datasets using Seurat^19^, so to individuate, if any, the characteristic features that discriminate “proper” oncogenes from randomly picked genes.

## Results

We explored several RNA-seq cancer datasets of bulk (population-based) and single cells that are available in the National Center for Biotechnology Information (NCBI)’s Gene Expression Omnibus (GEO) database^20^. We selected seven cancer types for bulk (breast^21^, colorectal^22^, leukemia^23^, liver^24^, ovarian^25^, skin^26^, and osteosarcoma^27^) and three types for single cells (breast^15^, ovarian^28^, and glioblastoma^29^) that are suitable for our analyses (see “Methods” for details). For proteomics assembly data, we searched for data on Proteomic Data Commons (PDC)^30^ and identified suitable datasets for liver and ovarian cancers.

### Bulk transcriptome and proteome noise and correlation analyses

**Fig. 1:**
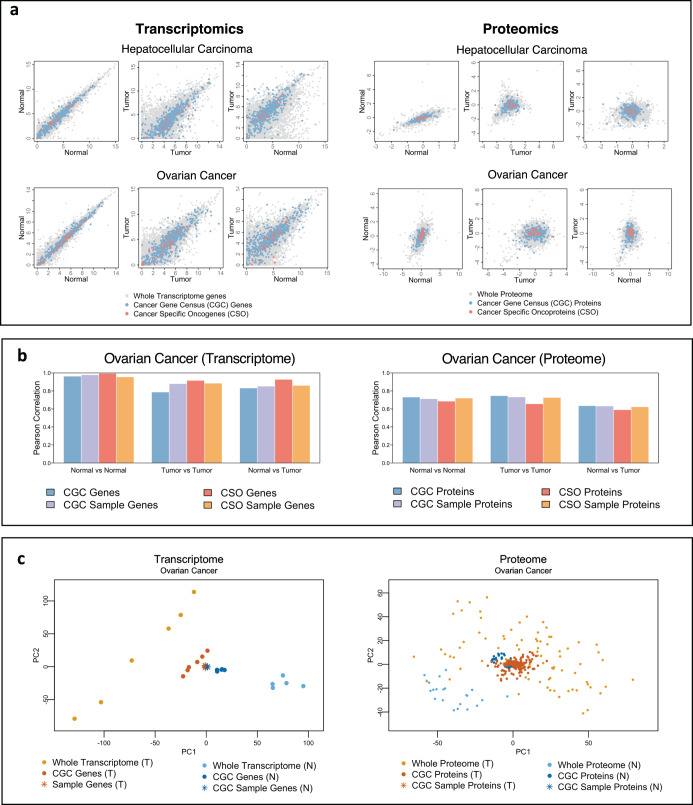
Expression invariance of oncogenes. Various types of statistical analysis were performed on bulk datasets to show the expression invariance of cancer genes. The analysis for transcriptomics samples is on the right panels, and the analysis for the proteomics samples is on the left panels. **a** Scatterplots between normal samples, tumor samples, and normal vs tumor samples for liver cancer and ovarian cancer, with the remaining types presented in Supplementary Fig. 1. Regular genes are represented by gray dots, CGC genes by blue dots, and CSO genes by red dots. **b** Pearson correlation for the expression levels of CGC genes (blue), CSO genes (red), CGC-sized sampled random genes (purple), CSO-sized sampled random genes (orange). Ovarian cancer was selected as an example for both proteome and transcriptome here, and the rest of the cancer types are in Supplementary Fig. 2. **c** PCA plots for whole dataset normal samples (light blue circles), whole dataset tumor samples (light orange circles), CGC genes normal samples (dark blue circles), CGC genes tumor samples (dark orange circles), CGC-sized random sampling of genes from normal samples (dark blue stars), CGC-sized random sampling of genes from tumor samples (dark orange stars), and the rest of the cancer types are in Supplementary Fig. 4.

Initially, we focused on bulk datasets. After performing quality control checks and lower expressions filtering (“Methods”), we investigated the level of global gene expression correlation (and, consequently, the relative amount of explained and stochastic variability). We compared transcriptome- and proteome-wide scatterplots of normal, cancer, and normal versus cancer sample pairs, and evaluated their corresponding Pearson (linear continuous), Spearman (monotonic rank-based) correlations, mutual information (MI, nonlinear dependence), and noise (square of the coefficient of variation^31^) (Table 1, Fig. 1a and Supplementary Fig. 1, gray dots). In general, as expected, the transcriptome-wide variability and noise are lower (and thus, correlation is higher), between normal samples when compared to between cancer samples or between cancer and normal samples (Fig. 1a, left panel). While a similar pattern can be observed in the proteomic data, the difference between cancer and normal samples in the expression of cancer genes is more pronounced (Fig. 1a, right panel).

**Table 1:** Transcriptome-wide and proteome-wide correlation values and noise.

Cancer type	R			$\rho$			MI			${\eta }^{2}$		
	N vs N	T vs T	N vs T	N vs N	T vs T	N vs T	N vs N	T vs T	N vs T	N vs N	T vs T	N vs T
Breast	0.967	0.947	0.941	0.867	0.87	0.829	0.727	0.792	0.632	0.321	0.304	0.363
Colorectal	0.964	0.928	0.936	0.837	0.812	0.793	0.697	0.6	0.564	0.583	0.63	0.656
AML	0.965	0.872	0.88	0.911	0.847	0.833	0.982	0.72	0.664	0.175	0.311	0.279
HCC	0.847	0.674	0.685	0.937	0.795	0.807	1.059	0.567	0.609	0.177	0.373	0.353
Ovarian	0.898	0.756	0.735	0.972	0.819	0.735	1.268	0.555	0.423	0.124	0.477	0.537
Osteosarcoma	0.837	0.833	0.752	0.942	0.811	0.76	1.121	0.668	0.53	0.222	0.349	0.489
Skin	0.939	0.985	0.947	0.77	0.813	0.704	0.694	0.658	0.482	0.453	0.326	0.464
HCC (proteome)	0.476	0.227	−0.144	0.495	0.176	−0.177	0.211	0.086	0.079	6E + 07	1E + 06	9E + 06
Ovarian (Proteome)	0.426	0.465	0.262	0.433	0.439	0.218	0.13	0.155	0.056	3E + 05	2E + 05	1E + 06

Transcriptome-wide Pearson correlation (R), Spearman correlation ($\rho$), noise (${\eta }^{2}$), and Mutual Information (MI), between normal samples (N vs N), tumor samples (T vs T), and normal vs tumor samples (N vs T).

Next, we focused on cancer-associated or cancer genes (~600 CGC and ~20 cancer-specific oncogenes (CSO), “Methods”) and compared their noise and scatter (Fig. 1a and Supplementary Fig. 1, blue and red dots, respectively). Notably, for breast, colorectal, liver, ovarian, and skin cancer transcriptomes, we notice that both CGC and CSO genes have lower scatter and noise (Table 2, Fig. 1a (right panel), Supplementary Fig. 1a, c, e) compared to their whole transcriptome, especially between normal and cancer. This is contrary to expectations since these genes are generally mutated in cancers and their expressions are anticipated to be significantly altered when compared to normal^32^. On the other hand, at the proteome level, CGC and CSO genes show slightly higher noise and variability (Table 2 and Fig. 1a, left panel). To avoid any statistical biases induced by size variation between the whole and subset of cancer genes, we also sampled CGC and CSO size random genes/proteins with 100 times repeated sampling (Fig. 1b, Supplementary Fig. 2a–g, and Table 2). For both transcriptome and proteome, the Pearson correlation and noise analyses show higher correlations and lower noise between normal samples, and the opposite trend between cancers and between normal and cancer samples for all sampling sizes. Interestingly, the correlations between random and cancer genes (CGC and CSO) in normal and cancer conditions, is similar, and in certain cases such as the liver transcriptome and the ovarian transcriptome, the CSO show greater correlations than random samplings. These data suggest that the expression variability and correlations of cancer genes are generally invariant with respect of the whole genome or randomly selected genes.

**Table 2:** Noise of cancer genes and random samples of genes in transcriptome and proteome.

Cancer type	N vs N				T vs T				N vs T			
	CGC	CGC sample	CSO	CSO sample	CGC	CGC sample	CSO	CSO sample	CGC	CGC sample	CSO	CSO sample
Breast	0.267	0.325	0.248	0.32	0.232	0.305	0.204	0.303	0.306	0.366	0.294	0.363
Colorectal	0.464	0.585	0.451	0.586	0.51	0.631	0.48	0.632	0.528	0.658	0.501	0.658
AML	0.145	0.176	0.131	0.174	0.249	0.315	0.246	0.311	0.218	0.282	0.212	0.278
HCC	0.173	0.17	0.172	0.177	0.337	0.357	0.363	0.372	0.322	0.335	0.316	0.351
Ovarian	0.07	0.127	0.073	0.126	0.39	0.48	0.404	0.477	0.45	0.536	0.461	0.537
Osteosarcoma	0.203	0.257	0.175	0.223	0.293	0.37	0.296	0.351	0.413	0.529	0.588	0.493
Skin	0.423	0.451	0.454	0.453	0.28	0.33	0.271	0.327	0.419	0.466	0.392	0.465
HCC (Proteome)	15457	5E + 05	1256	4E + 05	1E + 06	3E + 05	1930	3E + 05	5E + 05	4E + 06	11016	1E + 06
Ovarian (Proteome)	2E + 06	4E + 05	33743	86705	81443	6E + 05	3126	1E + 05	25978	5E + 05	7910	3E + 05

Noise between normal samples (N vs N), tumor samples (T vs T), and normal vs tumor samples (N vs T) for CGC genes, CGC-sized random sampling of genes, CSO genes, and CSO-sized random sampling of genes.

To check mutual and nonlinear dependence between the samples, we investigated mutual information (MI, “Methods”) based nonlinear correlations for both cancer and random genes (Supplementary Fig. 3). Again, the results are inconclusive in that we could not generalize across the different cancer types whether random or cancer genes display a different degree of association. While these results cannot be adjusted for the tumor purity of cancer samples since not all the datasets provided this information, the purity of the tumor samples in the skin^26^ and liver^24^ cancer transcriptomic datasets were established to be adequate (>30% tumor cell fraction, and 0.821–0.905 purity index respectively). Notably, the invariance of cancer genes from random genes is even stronger for liver cancer samples.

Thus, by studying scatterplots, noise, linear, and nonlinear correlations, we could not conclude whether CGC or CSO, collectively, display different statistical properties with respect to similarly sized random samples of genes in both transcriptomic and proteomic data. To probe this result further, we next performed dimensional reduction using Principal Component Analysis (PCA) (“Methods”).

### Dimensionality reduction by PCA

Figure 1c and Supplementary Fig. 4 show the PCA solution in the first two dimensions accounting for the largest variance amount. For both transcriptome and proteome, we observed that normal samples (light blue circles) are located closer to one another, whereas their cancer counterparts (light orange circles) are more dispersed across the *x–y* space (Fig. 1c and Supplementary Fig. 4). This result indicates that the whole biological datasets (Fig. 1c, transcriptome-left panel, proteome-right panel) of normal replicates is less noisy than that for cancers, confirming the results of the bivariate correlation analyses above. However, when we analyzed the same metrics for cancer genes (CGC and CSO), they are less variable and closer to the metrics between normal (dark blue circles) and cancer (dark orange circles). To test whether this is due to the size or gene number effects, as above, we tested random gene samples of the same size in the normal tissue (dark blue stars) and the tumors (dark orange stars). Notably, the locations of the randomly selected samples are almost invariant to those of the cancer genes, in both omics’ levels. Furthermore, the mean Euclidian distance between the samples’ locations in whole datasets between normal and cancer is the highest when compared with that of cancer genes or random genes alone (Table 3).

**Table 3:** Euclidian distances between normal and tumor samples.

Genes	Whole	CGC	CGC-sized random sampling	CSO	CSO-sized random sampling
Distance	144.1	26.27	23.7	4.78	2.75

Average Euclidian distances between normal and tumor samples in dimensionality reduction plots (PCA).

Taken together, these results indicate that although normal and cancer counterparts have vastly different amounts of heterogeneity at the whole transcriptome and proteome scale, they are, however, very similar when only cancer and randomly sampled genes are taken into consideration.

### Sample similarity analysis

**Fig. 2:**
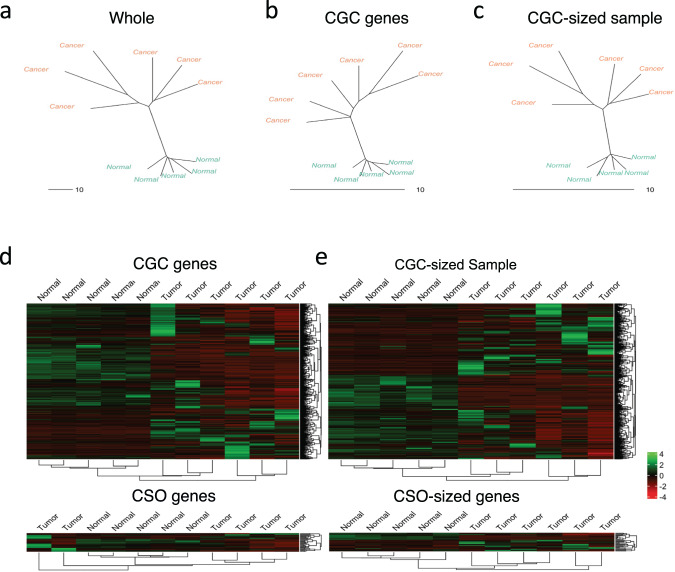
Sample Similarity Analysis. NJ sample tree of ovarian cancer (rest of cancer types shown in Supplementary Fig. 5) used to highlight the differences between tumor samples (orange) and normal samples (blue). The trees were generated from **a** whole transcriptome, **b** CGC genes, **c** CGC-sized random sampling of genes. Hierarchical clustering was used to show the differences between tumor samples and normal samples of ovarian cancer using **d** CGC genes and CSO genes, and **e** CGC-sized random sampling of genes and CSO-sized random sampling of genes.

To further investigate the observed invariance between CGC and random genes, we employed two types of sample similarity analyses on the transcriptomic datasets: Neighbor-Joining (NJ) and hierarchical clustering. We first used the whole transcriptomes to project samples from each cancer type into NJ dendrograms (Fig. 2a and Supplementary Fig. 5, left panels). For breast, glioma, ovarian, and osteosarcoma, the dendrograms show clear separations and clusters differentiating cancer (orange), and normal samples (blue). For the remaining leukemia, liver, and skin, the dendrograms, however, appeared randomly clustered for a subset of cancer and normal samples. This may not be surprising as some cancer data are highly heterogenous even between their replicates^33^.

Investigating the specific effect of CGC genes on NJ dendrograms, we observed that the overall distance between all samples decreased significantly, in a similar manner to our dimension reduction analysis (Fig. 2b and Supplementary Fig. 5, middle panels). However, it is worth noting the fact that for some cancers small local changes occurred where tumor samples were rearranged. To avoid the statistical bias induced by sample size variation, we once again performed the sample similarity analysis on a CGC-sized random sampling of genes. As anticipated, the distance between samples decreased for the random sampling of genes as well (Fig. 2c and Supplementary Fig. 5, right panels), with the overall mean sum of branch lengths between the samples being comparable. Unlike the CGC genes, however, the dendrograms generated from the random sampling of genes were highly similar to the whole transcriptome dendrograms (Fig. 2a, c and Supplementary Fig. 5, right and left panels). Unexpectedly, the dendrograms generated using the CGC genes had the tumor samples mildly rearranged. While these results also show invariance between CGC and randomly sampled genes when it comes to differentiating between tumor and normal samples, CGC genes do appear to play some role in among tumor sample differences.

Next, we performed hierarchical clustering for CGC, CSO and randomly sampled genes (Fig. 2d, e and Supplementary Fig. 6). Notably, only in some cancers CGC and random genes were able to correctly cluster cancer and normal samples (ovarian and osteosarcoma), while in the rest the clustering was not precise. Nevertheless, the overall clustering based on CGC and random genes were highly similar for all cancers with minor rearrangements only. In the case of CSO, the separation between normal and tumor samples decreased when compared to the CGC clustering in all cancer types. Furthermore, when we compared this outcome with the results of the CSO-sized sampling of random genes, we observed that the random genes performed poorly in separating tumor samples and normal samples due to the small number of genes.

Overall, these results point to the similar clustering of samples between CGC and CGC-sized random samplings of genes, as well as CSO and CSO-sized random samplings of genes, suggesting invariance as observed for the previous sections. Thus far, the analyses are unable to highlight any significant effect of CGC genes compared to the rest of transcriptome.

### PPI network analysis

Since the definition of oncogenes only allows for protein-coding genes, their interaction properties can be investigated to clarify whether oncogenes are truly like any other protein-coding genes in the transcriptome. Notably, biological PPI networks tend to display power law distribution^34^ with few nodes (hubs) having thousands of interactions (edges), while most nodes (leaves) will have a few or just a single interaction.

**Fig. 3:**
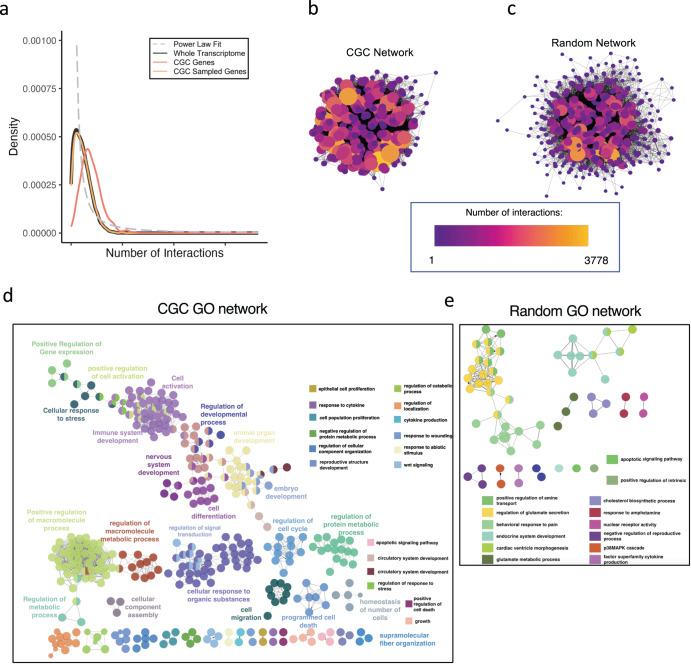
PPI and network analysis. **a** Using the literature-known connectivity properties of protein-coding genes we generated a density plot for the number of PPI per gene for all human protein-coding genes (black), CGC genes (red), CGC-sized random sampling of genes (yellow) and the fitted power law distribution (gray dashed line). We then used Cytoscape to visualize PPI interaction networks for **b** CGC genes and **c** CGC-sized random sampling of genes. We also explored the GO networks (“Methods”) generated from **d** CGC genes and **e** a set of randomly sampled genes of the same size as the CGC set.

We plotted the distribution density of the number of interactions per gene for all known protein-coding genes in STRING and GeneMania databases combined^35,36^ (black), and found it to follow the general trend of the power law distribution (dotted gray line), where only a strict minority of genes have several thousand of interactions followed by a rapid decrease in connectivity for the other genes (Fig. 3a). Next, we investigated the PPI distributions of CGC and CGC-sized random genes. First, for CGC-sized random genes (yellow), it can be observed that the distribution of interactions looks similar to that of the whole, except for the peak density, which is slightly lower. Second, for the distribution of PPI per CGC gene (orange), we observe that its mean is higher, and the density plot is shifted further from the power law curve (gray). When computing the average number of interactions per gene, we observed that the whole transcriptome and the random sampling have a nearly identical average, whereas the CGC genes have a significantly higher mean number of interactions per gene (Table 4). This result is not surprising since cancer genes are much more widely studied with respect to all other genes, so their number of connections (stemming from literature data) is likely to be higher. Furthermore, some cancer genes are known to be transcription factors (TFs) as well, which would explain their connectivity. Nevertheless, only 20% of CGC genes (Supplementary Fig. 7) are found to be TFs^37^, and upon removing those 20%, the average number of PPI does not drop significantly (Table 4). Thus, the results highlight CGC oncogenes as a special subset of genes with above-average connectivity and much more homogeneous (with respect to the whole gene set) in terms of their physiological roles.

**Table 4:** Mean number of interactions.

Gene set	Average number of PPI interaction
Whole	1115.963
CGC	1841.178
CGC—TF genes	1798.995
Random sample	1114.693

Mean number of PPI interactions for whole transcriptome, CGC genes, random samplings of genes, and subset of CGC genes that does not include any CGC TF genes.

To further illustrate this point, we generated PPI network plots for CGC and random genes (Fig. 3b, c). A color and size gradient were used to highlight the number of interactions per node, where larger connectivity shows bigger size and lighter color. When compared to the randomly sampled genes, the CGC network possesses more highly connected nodes, similar to network hubs, and fewer nodes with single connections. Furthermore, the CGC network appeared to have a significantly higher number of edges connecting the nodes, indicative of CGC genes being genes related to each other. The random network, on the other hand, despite containing genes with a relatively high number of global connections, appeared to be considerably less locally connected.

Lastly, in order to investigate the biological significance of CGC genes, we generated GO networks using ClueGO^38^ in Cytoscape (Fig. 3d). We observed that the GO network of CGC genes is dense and composed of various crucial biological processes that can affect cell fate. On the other hand, when we generated GO networks from the same number of random genes as CGC genes (Fig. 3e), we observed that the network is sparser with fewer biological terms that are able to pass the minimum filtering threshold. Lastly, unlike in the CGC GO network, where each node was composed of tens to hundreds of genes, in the random GO network the maximum number of genes per node is 6 genes. This shows that CGC genes cover a wide range of interconnected biological processes that have the ability to affect cell fate.

Together, these results emphasize the distinctiveness or importance of oncogenes as the more highly connected “hub genes” and suggest that the insignificant behavior of oncogene expression levels in transcriptomic data might not be reflective of their true importance.

### scRNA-seq transcriptome analysis

**Fig. 4:**
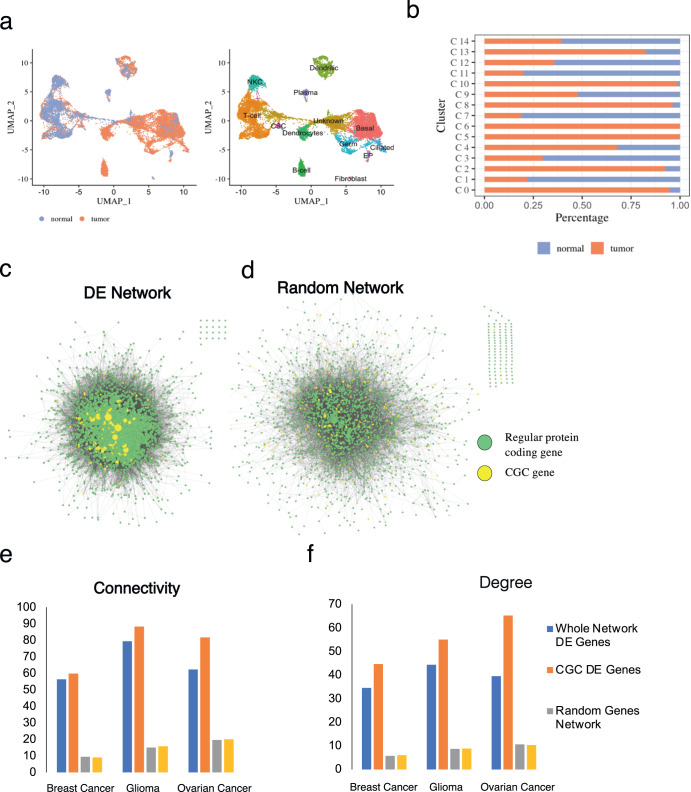
scRNA-seq analysis and network properties of oncogenes. **a** uMAP dimensional reduction for integrated tumor and normal samples of ovarian cancer where different colors represent different cell populations. **b** Cell condition composition breakdown per cluster in ovarian cancer. DE analysis identified DE genes between normal and tumor cells in each cluster. These DE genes were used in Cytoscape to generate PPI networks for **c** DE network and random network of the same size from **d** random sampling of genes for comparison. In the PPI networks, node size scaled to the degree of the gene, and highlighted the CGC genes (yellow). The average **e** connectivity and **f** degree properties of the whole DE network (blue), and CGC DE genes (orange) are higher when compared to the random network (gray), and the random subset of genes (yellow).

The advantage of single-cell sequencing is the fact that it offers a more in-depth overview of individual cell expression levels within various subpopulations, which is essential considering the complex nature of tumor microenvironments that employ a wide array of cell types during tumor progression. Therefore, to investigate the expression patterns of oncogenes within various cell populations we searched the GEO database for scRNA-seq datasets that contained patient tumor and normal samples. We selected three scRNA-seq datasets from the GEO database (“Methods”) composed of paired normal and tumor patient tissue samples for breast cancer, ovarian cancer, and glioma. We first performed quality control and normalization, after which we proceeded to integrate normal and tumor samples for concurrent analysis of cancer gene expression (“Methods”). We observed a diverse microenvironment (Supplementary Tables 3–5) in all three cancer types (Fig. 4a and Supplementary Fig. 8a, c, g), with each cluster comprising different proportions of cancer and normal subpopulations (Fig. 4b and Supplementary Fig. 8b, d).

To verify whether there exist cancer-specific differences in gene expressions at each cluster, we performed Differential Expression (DE) analysis between tumor and normal cells of the preserved clusters across the two conditions (e.g., cluster 9, Fig. 4b). Our analyses show that 5–10% of all the identified DE genes in every cluster and across all three cancer types are CGC genes (Table 5). Amongst the differentially expressed CGC genes (Table 6), we observed and visualized several oncogenes that have been previously studied for their role in different cancers (e.g., *TMSB4X* and *TNFAIP3*, Supplementary Fig. 9). Furthermore, despite representing only a small fraction of the network, CGC genes also exhibit special connectivity properties (“Methods”) within the network of DE genes (Fig. 4c, e, f). To avoid sample size bias in our results, we similarly generated random networks composed of the identical number of nodes as the DE network (Fig. 4d). For these random sets of genes of identical size, the differentially expressed DE genes exhibit average connectivity properties similar to that of the larger networks, that is, of lower connectivity compared to CGC genes. (Fig. 4e, f). This result is consistent with our previous analysis, and with the fact that CGC genes tend to be better connected due to them being better studied in the literature.

**Table 5:** Number of identified DE genes.

Type:	DE genes	CGC
Breast cancer	1249	72
Glioma	1866	115
Ovarian cancer	2240	166

The number of DE genes identified in each cancer type and the number of CGC DE genes.

**Table 6:** DE CGC genes for each cancer type.

Type	DE Genes
Glioma	ABL1, ABL2, AFDN, ALDH2, ARHGEF12, ATP1A1, B2M, BAX, BAZ1A, BCL3, BTG1, CALR, CASP3, CBL, CCND1, CD74, CDH11, CHCHD7, CHST11, CLTC, COL1A1, COL3A1, COX6C, CREB3L2, CTCF, CTNNB1, CUL3, DNM2, ETV6, EXT1, EZR, FGFR1, FLNA, FLT4, FNBP1, GNAQ, H3F3A, HERPUD1, HEY1, HIF1A, HLA-A, HRAS, HSP90AB1, IDH1, IDH2, ITGAV, JAK1, KDM5A, KDR, KLF4, KLF6, LATS2, LCP1, LMNA, LYN, MAFB, MALAT1, MALT1, MAP3K13, MAPK1, MSI2, MSN, MYH9, MYO5A, NACA, NFE2L2, NFKBIE, NPM1, NR4A3, PABPC1, PDE4DIP, PDGFB, PPP2R1A, PRDM1, PREX2, PTPRK, RAC1, RALGDS, RANBP2, RAP1GDS1, RB1, REL, RHOA, RPL10, RPL22, RPL5, RPN1, SET, SFPQ, SGK1, SH2B3, SH3GL1, SIRPA, SMARCA4, SND1, SOCS1, SRSF3, TET2, TFPT, TMEM127, TMSB4X, TNFAIP3, TOP1, TP53, TPM3, TPM4, TRAF7, TRIM24, USP9X, WAS, WWTR1, YWHAE, ZEB1, ZNF331
Ovarian	WWTR1, CD74, BIRC3, BCLAF1, CDH1, TPR, ARID1B, THRAP3, CASP8, ERBB3, KLF6, CBFB, PABPC1, TFRC, AFF4, FCGR2B, SDHA, KDM5A, IGF2BP2, MAP3K13, HSP90AA1, PTPRC, XPO1, CYLD, ATRX, MECOM, BAX, GNAS, FUS, FH, EZR, HSP90AB1, MYH9, HIF1A, ARHGAP5, TSC2, NDRG1, UBR5, TFPT, CCDC6, RNF43, DDX5, PRKAR1A, BRCA1, YWHAE, CCND1, PPFIBP1, CHD4, QKI, CBLB, SF3B1, MSH6, SFPQ, TNFAIP3, SGK1, CCND2, SET, DNMT3A, BCL11A, CXCR4, HNRNPA2B1, ACSL3, USP9X, DEK, PAX8, KDM5C, KTN1, MAP2K2, SMARCA4, AKAP9, HIP1, SPECC1, AFDN, CLIP1, DNAJB1, H3F3B, CDC73, LCP1, RAC1, ABI1, HMGA1, NUMA1, ATIC, IDH1, ITGAV, USP8, TCF12, PML, ZFHX3, NCOR1, CLTC, SDHC, TPM3, EML4, LPP, ARHGAP26, NONO, NFIB, CDKN2A, RANBP2, MSI2, GNAQ, EIF4A2, SLC34A2, RUNX1, LMNA, IKZF3, SRSF2, FUBP1, REL, H3F3A, ATP1A1, ELF3, RPN1, PBRM1, RAD21, COX6C, NSD1, B2M, STAT6, TPM4, CTNNB1, STAT3, ASPSCR1, CNBP, CAMTA1, BCL2, EIF1AX, RNF213, DDIT3, SMAD2, TBL1XR1, JUN, PDE4DIP, CALR, MUC16, NPM1, SETD2, IDH2, CREB3L2, CSF1R, MAML2, WT1, SOCS1, MUC1, PBX1, IKZF1, SRGAP3, NCOR2, NF1, FLNA, SND1, TRIM33, SIRPA, CTNND1, MDM4, TOP1, TRIM27, TMSB4X, HLA-A, DDX3X, ETV5, MALAT1, LSM14A, CUX1, NCOA4
Breast	AKAP9, ATP1A1, B2M, BTG1, BTG2, CALR, CAMTA1, CCND1, CD74, CD79A, CDH11, CDKN1A, CDKN2A, CDKN2C, CNBP, COL1A1, COL3A1, COX6C, CTNNB1, DDX5, DEK, DNAJB1, EIF1AX, EIF3E, EIF4A2, ELF3, EPAS1, EZH2, FUS, GATA3, GMPS, GNAS, HERPUD1, HLA-A, HSP90AA1, ID3, IDH2, JUN, KDSR, KLF6, LHFPL6, LMNA, MAFB, MALAT1, MAP3K13, MYH9, NDRG1, NFIB, NONO, NSD3, PABPC1, PBX1, PCBP1, PDGFRB, PSIP1, RAC1, RAD21, RHOA, RPL10, RPL22, RPN1, SDC4, SDHB, SDHC, SET, SMARCA4, TCEA1, TFG, TMSB4X, TNFRSF17, TPM3, TPM4

CGC DE genes between normal and tumor cells from the same cluster for each cancer scRNA-seq dataset.

Taken together, these results show indeed that there exist notable differences between normal and tumor tissues that emerge on a single-cell level from the heterogeneous tumor microenvironment (reflected by cell clusters). Differential expression between normal and tumor cell types in cell clusters, as well as the enhanced connectivity properties of cancer genes highlight the fact that oncogenes can be distinguished from other genes as a special subset.

Together, the analysis of single-cell transcriptomic data revealed not only a heterogeneous tumor microenvironment (reflected by cell clusters), but also heterogeneous expression patterns for oncogenes that cannot be generalized on a global population level. Furthermore, differential expression analysis between similar cell clusters in tumor and normal samples further highlighted the fact that oncogenes can be distinguished from other genes as a special subset with distinct network connectivity properties.

## Discussion

Cancer is a highly complex and variable disease. It is, thus, conceivable that the variability among cancer patients in terms of gene expression patterns (e.g., transcriptomics, proteomics) will be large. Correspondingly, in Fig. 1a and Supplementary Fig. 1, we show the transcriptome-wide scatter (right panel) and proteome-wide scatter (left panel) both showing a significant amount of noise between cancer samples or between normal versus cancer samples, even in the presence of a relevant correlation corresponding to the presence of a “tissue-specific” attractor. Nevertheless, when focusing on elements that are known as cancer genes or oncogenes (here noted as CGC and CSO genes), we observe lower variability and noise with respect to oncogenes-sized random sets of genes, indicating a more correlated behavior of oncogenes between normal and cancer or between cancer samples. The same behavior is apparent when correlation, mutual information and dimensional reduction using PCA were performed (Fig. 1c, d and Supplementary Figs. 2–4). Furthermore, sample similarity (nj-tree and hierarchical clustering heatmap) analyses show almost identical clustering between oncogenes and random gene samples (Fig. 2 and Supplementary Fig. 5), again pointing to invariance. Thus, the global behavior of oncogenes at this very general level, cannot be neatly separated by the behavior of the rest of both transcriptome and proteome. On the other hand, when the analysis focuses on specific distributional feature, the much higher internal consistency of oncogenes expression levels clearly emerges, this increased consistency, has both a methodological and a biological origin. The methodological has to do with the well-known difference between correlation and causation^39^. The difficulty of setting up an experiment allowing for a clear discrimination between correlation and possibly causative factors in biology is linked to the large circular causality of complex systems^40^. We believe this methodological weakness is causing severe biases in current cancer research.

The biological origin of the ambiguity, ending up in the paradoxical but statistically consistent statement that almost any gene is a cancer gene^17^, stems from the fact that genes do not work in isolation but as coherent modules. This implies that any variation that derives from specific “driver” genes reverberates across the whole genome expression, putting the entire set of expressions on another state or “attractor” mode. This is evident in Fig. 3, where we observe that the oncogenes clearly possess better connectivity (leading to more network hubs^41^) compared to the random selection of genes, regardless of whether they are TF genes or not. That is, the oncogenes tend to generally act as “hubs” of interaction networks and, thus, the “relevance for cancer” is not an essential property of single genes but an emergent property of groups of genes where oncogenes play a major role at the bulk scale^42^. This makes inter-module switch genes that mediate the relation between different parts of the biological regulation network affine to “causative” agents^43^.

Finally, when moving into single-cell scale, we can observe that a subset of oncogenes show specific behavior at cell cluster levels, representing subpopulations of cells (Fig. 4). In other words, only when shifting to single-cell scale we start to appreciate significant expression differences between oncogenes and random gene collections. Notably, the relevance of cancer subpopulations heterogeneity is now being recognized by newer dynamical approaches to cancer research^44,45^. This heterogeneity can be fully appreciated only by the adoption of single-cell level analysis^46^, and this is perhaps why only in the case of single-cell RNA-seq studies we are able to uncover putative and possibly “cancer driver” genes that are totally unnoticeable at the bulk level.

In summary, we can state that the accumulation of huge amount of molecular data made evident the need for a radical “change of scale” of cancer research. This change of scale is analogous to the rise of statistical mechanics-inspired methods in physics and implies the shift from linear causative paths at single gene level to the search for general organization principles of strongly internally correlated systems^47^. Our work highlights the fact that oncogenes need to be reviewed in the context of association and causality, as it is easy to show that any gene can be highly correlated with cancer genes. Nevertheless, when one take’s a network view, oncogenes show higher connectivity than random genes, In the future, what constitute an oncogene needs to be deeply scrutinized by adopting protein causality studies and higher-resolution single cells analyses.

## Methods

### Oncogene gene sets

The oncogene gene sets were obtained from the Cancer Gene Census (CGC) of the Catalog of Somatic Mutations In Cancer (COSMIC) database^14^. Cancer-specific oncogenes were defined using the tumor type identifiers provided by the database and are listed in Supplementary Table 1.

### Bulk proteomics datasets and pre-processing

Bulk proteomics datasets were downloaded from the Proteomic Data Commons (PDC)^30^ from previously published datasets for liver cancer (PDC00198), and ovarian cancer (PDC00010). No missing value imputation was performed, and all genes with missing values were removed (~20%). The exact number of genes and samples considered for the analysis are listed in Supplementary Table 2.

### Bulk RNA-seq datasets

Bulk RNA-seq datasets were downloaded from the Gene Expression Omnibus (GEO) database from previously published data for eight cancer types: breast (GSE183947)^21^, colorectal cancer (GSE165255)^22^, acute myeloid leukemia (GSE138702)^23^, hepatocellular carcinoma (GSE112705)^24^, high grade serous ovarian cancer (GSE190688)^25^, osteosarcoma (GSE126209)^27^, oral squamous cell carcinoma (GSE184616)^26^. The datasets were chosen based on the availability of patient tumor samples as well as paired patient healthy tissue samples. Only relevant samples from each dataset were selected. The datasets were all converted to transcripts per million (TPM) counts for consistency. All the replicates are considered to be biological replicates (i.e., referring to independent individuals), and not as technical replicates.

### Single-cell datasets

Single-cell RNA-seq datasets were downloaded from the GEO database for three cancer types: breast (GSE161529)^15^, ovarian (GSE181955)^28^, glioblastoma (GSE162631)^29^. For all three datasets, only a subset of samples was used.

### Statistical distribution fitting

For each sample in the bulk RNA-seq datasets, the TPM expression values were fitted to theoretical statistical distributions such as log-normal, Pareto, Burr, loglogistic, and Weibull^48^. The best-fitted distribution for each sample was selected based on the minimum Akaike information criterion value, and approximate thresholds for filtering were selected based on how closely the experimental distributions follow the theoretical distributions^49^. The *fitdistrplus* package^50^ was used for parameter estimation, and the *mass* package^51^ for the aforementioned theoretical distributions.

### Correlation analyses

For all datasets, correlation for a set of *n* genes was computed between two samples of interest. The total mean correlation value was then calculated by finding the average of all correlation values.

#### Pearson correlation

The Pearson correlation coefficient between two samples (vectors X and Y) for a set of *n* genes can be defined as:

1 $$r\left(X,Y\right)=\,\frac{{\sum }_{i=1}^{n}\,\left({x}_{i}-\,{\mu }_{X}\,\right)(\,{y}_{i}-\,{\mu }_{Y}\,)}{{\sigma }_{X}{\sigma }_{Y}}$$

where *x*_*i*_ and *y*_*i*_ are the expression value for the *i*th gene in the dataset for each of the two samples. Similarly, ${\mu }_{x}$ and ${\mu }_{y}$, represent the average expression of each sample, and the standard deviation of the expression values in each respective sample.

#### Spearman correlation

Similar to the Pearson correlation, Spearman rank correlation is defined by:

2 $$\rho \left(X,Y\right)=1-\,\frac{{\sum }_{i=1}^{n}\,{({r}_{x,i}-{r}_{y,i})}^{2}}{n(\,{n}^{2}-1\,)}$$

Where *r*_*x*,i_ and *r*_*y,i*_ are the ranks of the *i*th gene in the two samples of interest.

#### Mutual information

The nonlinear dependency between two samples can be checked by mutual information:

3 $$M\,I(X,Y)=-\mathop{\sum }\nolimits_{i=1}^{M}p{({x}_{i})}{{\ln}}(p({x}_{i}))-\mathop{\sum }\nolimits_{i=1}^{M}p({y}_{i}){\mathrm{ln}}{\left(\right.p({y}_{i})}+\mathop{\sum }\nolimits_{i=1}^{M}p(x,y){\mathrm{ln}}(p(x,y))-\varepsilon$$

where the joint probability distribution function *p(x,y)* and marginal probability distribution functions are estimated by discretizing the rank-transformed gene expression into bins. For each transcriptome, the number of bins used in the analysis was computed using Doane’s rule^52^. The MI between samples was computed using the *infotheo* package^53^.

### Average noise analysis

For a set of *n* genes, the total average noise between two samples can be defined as:

4 $${\eta }_{{tot}}^{2}=\frac{1}{n}{\sum }_{i=1}^{n}{\eta }_{{iXY}}^{2}$$

where the noise of the *i*th gene between two samples of interest is defined as ${\eta }_{{iXY}}^{2}$ and can be calculated by dividing the variance (${\sigma }^{2}$) by the squared mean expression (${\mu }^{2}$):

5 $${\eta }_{{iXY}}^{2}=\frac{{\sigma }_{{iXY}}^{2}}{{\mu }_{{iXY}}^{2}}$$

### Neighbor-joining tree

The *ape*^54^ package was used to compute dendrograms for all samples using the neighbor-joining (**nj**) algorithm. For every dataset composed of *m* samples and *n* genes, the *nm* matrix of expression values was used to compute a distance matrix between the *m* samples using the *stats* package^55^. The algorithm assumes a star network and uses the distance matrix to build the dendrogram into a completely resolved unrooted tree with known branch lengths. In the final tree, samples that are more similar cluster together and are separated by shorter overall branch lengths.

### scRNA-seq analysis

#### QC and normalization

All scRNA-seq datasets were analyzed using the *Seurat* package^19^. Every dataset was filtered out for cells based on the following QC metrics: number of unique genes per cell, and percentage of mitochondrial counts. Only cells with over 200 unique genes per cell and less than 15% mitochondrial counts were retained. The data were then normalized using the “LogNormalize” method provided by the *Seurat* package, which normalizes the count values for each cell by the total counts for the cell and multiplies it by a scale factor of 10000.

#### Integration

Top 2000 variable features for every sample were selected using the “*vst”* method in the *Seurat* package for further analysis. The method aims to account for the mean-variance relationship in scRNA-seq that arises due to technical factors by fitting a polynomial regression to the relationship between the log(variance) and log(mean) of every gene. This allows for the computation of standardized variances that are used to rank the genes directly.

Based on the number of samples they are deemed variable in, the list of variable features is further reduced to the top 2000 genes that have the highest variability across most samples. Then, cells that can serve as integration anchors across the different samples are selected using the *FindIntegrationAnchors* that is described in the *Seurat* package. Lastly, based on the identified integration anchors, and ranked list of variable features, the samples were integrated, and the expression values were corrected such that the finalized integrated dataset can be treated as a single normalized dataset. The final integrated dataset from each cancer type was then used to identify the composition of the samples. The dataset was first scaled on feature-level using the standard deviation of the expression of each gene such that the expression will be centered to have a mean of zero. The scaled dataset was used to perform PCA as an intermediate step in the dimension reduction analysis. Similarly, uMAP was then performed to embed the single cells into the Euclidian space. Lastly, the shared nearest-neighbor (SNN) clustering algorithm was used to identify individual cell clusters.

#### Cell marker identification and annotation

Top five biomarkers were selected for every identified cluster with the *FindAllMarkers* function using the Wilcoxon rank-sum test. We then used CellMarker^56^ and PanglaoDB^57^ to predict the cell type based on the top five biomarkers.

#### Differential expression

Using the clusters identified from the integrated analysis, we performed differential expression between tumor cells and normal cells classified into the same cluster. The *FindMarkers* function was used to find differentially expressed genes that were expressed in at least 50% of the cells of interest.

### PPI data

The protein–protein interaction (PPI) data were obtained from STRING and GeneMania databases^35,36^. The protein identifiers of the two datasets were converted to UniProt accessions. Then we merged the two datasets and removed any redundant interactions, keeping a single instance of each interaction.

### Cytoscape network analysis

Networks using DE genes were generated using the Cytoscape StringApp^58,59^. The software’s Analyzer^60^ was used to analyze the network and compute the degree parameters for each gene in all the networks. The parameters considered were degree which refers to the number of connections per node, as well as connectivity, which refers to the number of connected components in the subset of nodes, where a higher value indicates a better-connected network, and lower values indicate elements that are more disjoint and poorly connected^60^.

#### ClueGO

GO Networks were generated in Cytoscape for Biological Processes only. The network specificity was chosen to be global, and the significance threshold was set to 0.05.

### Reporting summary

Further information on research design is available in the Nature Research Reporting Summary linked to this article.

## Supplementary information

Supplementary Information

Reporting Summary

## Data Availability

All data needed to evaluate the conclusions in the paper are present in the paper and/or the Supplementary Materials.
