# Supplementary material for: Globally invariant behavior of oncogenes and random genes at population but not at single cell level
Source: NPJ Syst Biol Appl. 2023 Jun 24;9:28. doi: 10.1038/s41540-023-00290-9 (PMC10290669; doi:10.1038/s41540-023-00290-9)
Supplement: Supplementary file 1 — Supplementary Information [file 41540_2023_290_MOESM1_ESM.pdf]

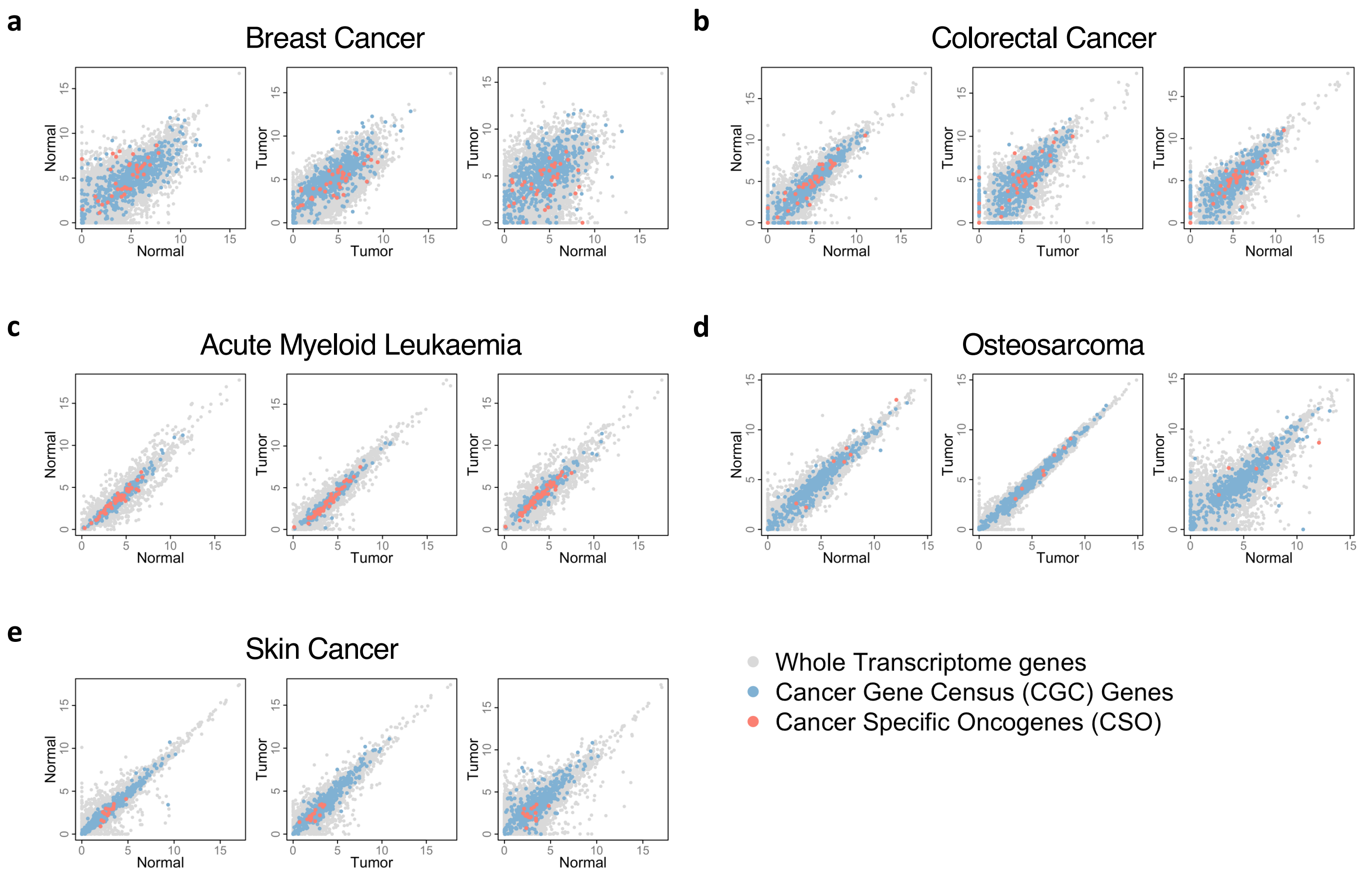

Supplementary Figure 1. Transcriptome-wide scatterplots for various types of cancer. Scatter plots for **a)** breast cancer, **b)** Colorectal cancer, **c)** Acute Myeloid Leukemia, **d)** Osteosarcoma, **e)** Squamous Cell Carcinoma between normal samples, tumor samples, and normal vs tumor samples three selected cancer types. All genes are represented by grey dots, CGC genes by blue dots, and CSO genes by red dots.

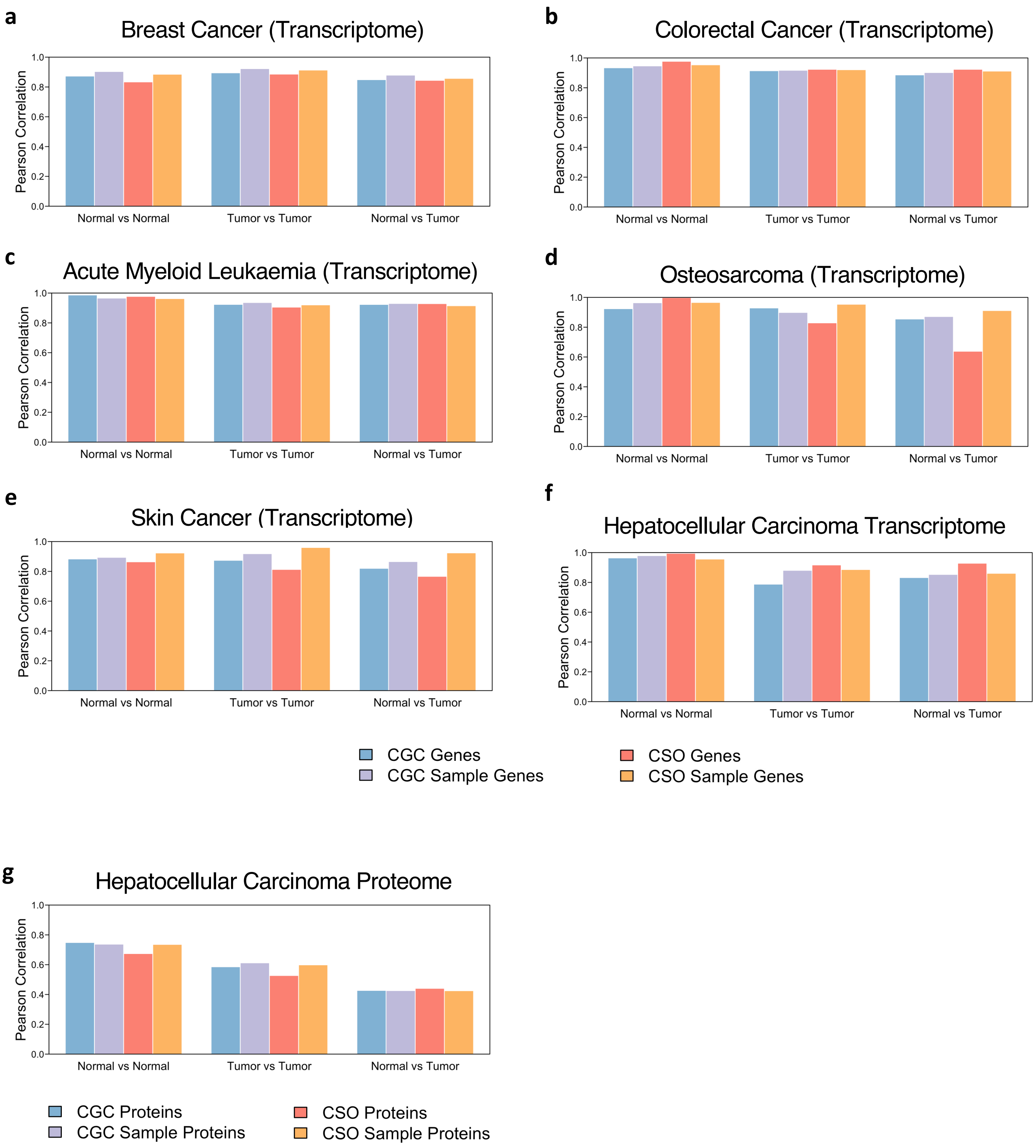

Supplementary Figure 2. Pearson Correlation between sample types for selected cancers

Pearson correlation between sample types for the expression levels of CGC genes (blue), CSO genes (red), CGC-sized sampled random genes (purple), CSO-sized sampled random genes (orange) for the transcriptome of **a)** breast cancer, **b)** colorectal cancer, **c)** AML, **d)** Osteosarcoma, **e)** HCC, and the proteome of **g)** HCC.

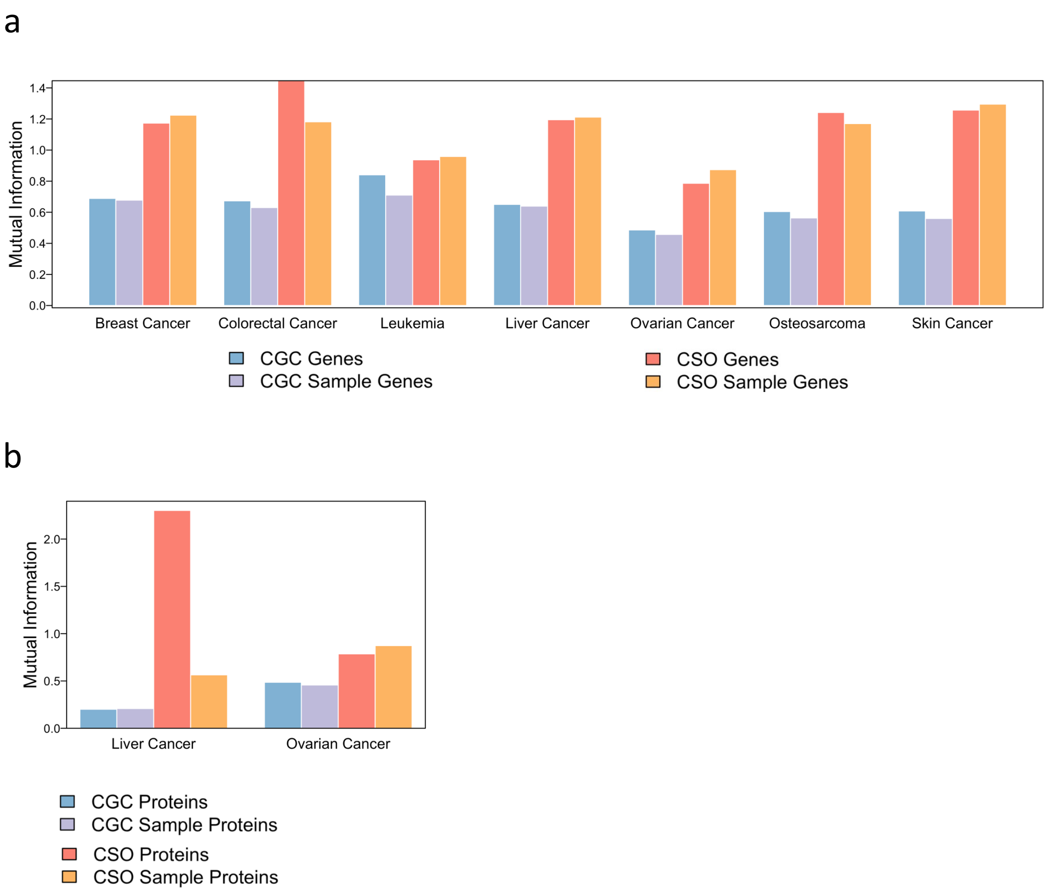

Supplementary Figure 3. Mutual Information of transcriptome and proteome cancer genes. Mutual Information between sample types for the expression level of CGC genes (blue), CSO genes (red), CGC-sized sampled random genes (purple), CSO-sized sampled random genes (orange) for **a)** transcriptomic samples, and **b)** proteomic samples**.**

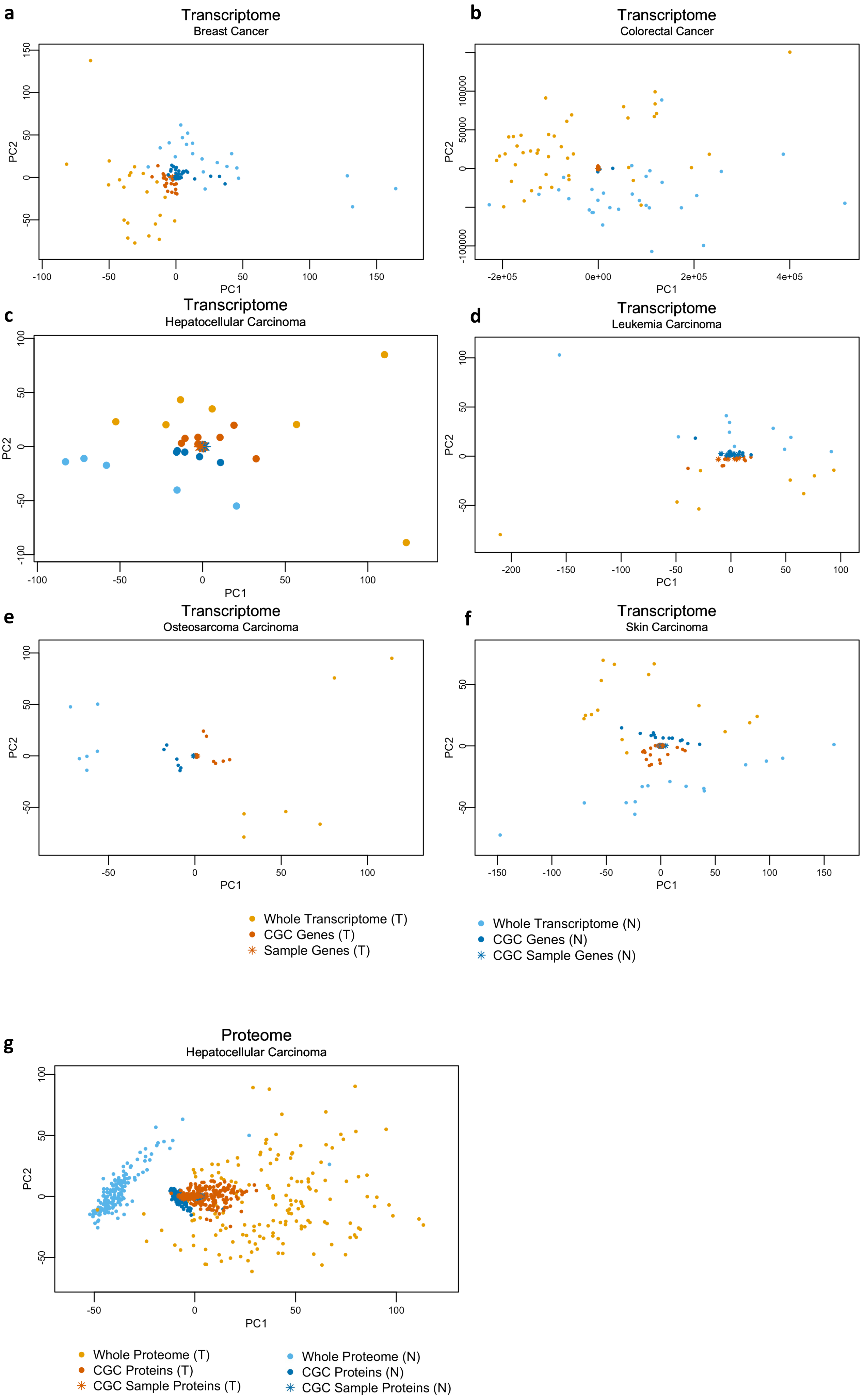
a
Transcriptome
Breast Cancer
PC1
PC2
b
Transcriptome
Colorectal Cancer
PC1
PC2
c
Transcriptome
Hepatocellular Carcinoma
PC1
PC2
d
Transcriptome
Leukemia Carcinoma
PC1
PC2
e
Transcriptome
Osteosarcoma Carcinoma
PC1
PC2
f
Transcriptome
Skin Carcinoma
PC1
PC2
Whole Transcriptome (T)
CGC Genes (T)
Sample Genes (T)
Whole Transcriptome (N)
CGC Genes (N)
CGC Sample Genes (N)
g
Proteome
Hepatocellular Carcinoma
PC1
PC2
Whole Proteome (T)
CGC Proteins (T)
CGC Sample Proteins (T)
Whole Proteome (N)
CGC Proteins (N)
CGC Sample Proteins (N)

Supplementary Figure 4. Tumor and normal samples in the dimension reduction space. PCA plot for whole transcriptome normal samples (light blue circles), whole transcriptome tumor samples (light orange circles), CGC genes normal samples (dark blue circles), CGC genes tumor samples (dark orange circles), CGC-sized random sampling of genes from normal samples (dark blue stars), CGC-sized random sampling of genes from tumor samples (dark orange stars) in for the transcriptomes of **a)** breast cancer, **b**) colorectal cancer, **c)** liver cancer, **d)** leukemia, **e)** osteosarcoma, and **f)** skin cancer and proteome of **g)** liver cancer.

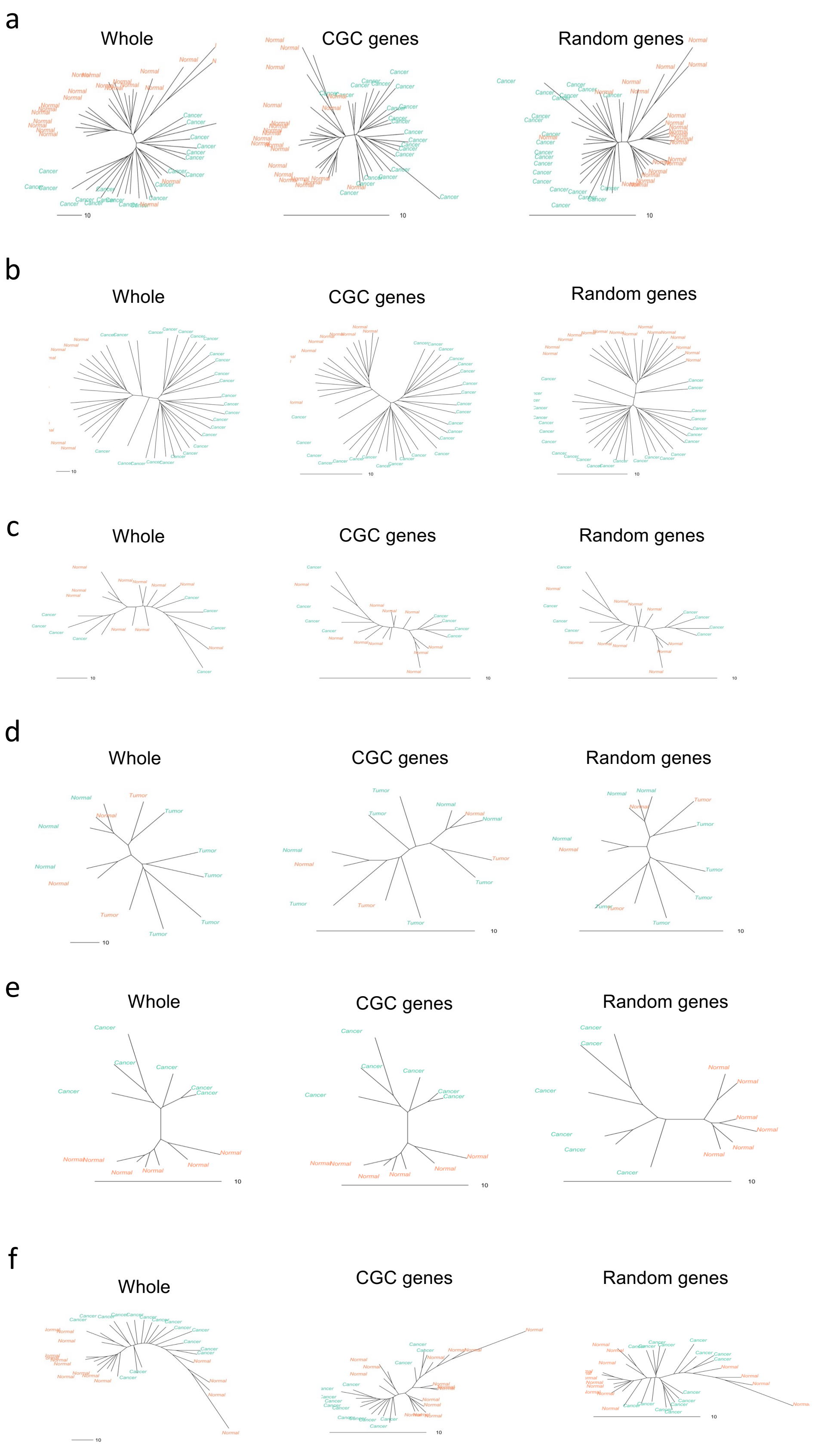

Supplementary Figure 5. NJ trees for tumor and normal samples for selected cancer types. NJ trees generated form the whole transcriptome, CGC genes and a random sampling of genes in **a)** breast cancer, b) colorectal cancer, **c)** leukemia, **d)** liver cancer, **e)** osteosarcoma, and **f)** skin cancer.

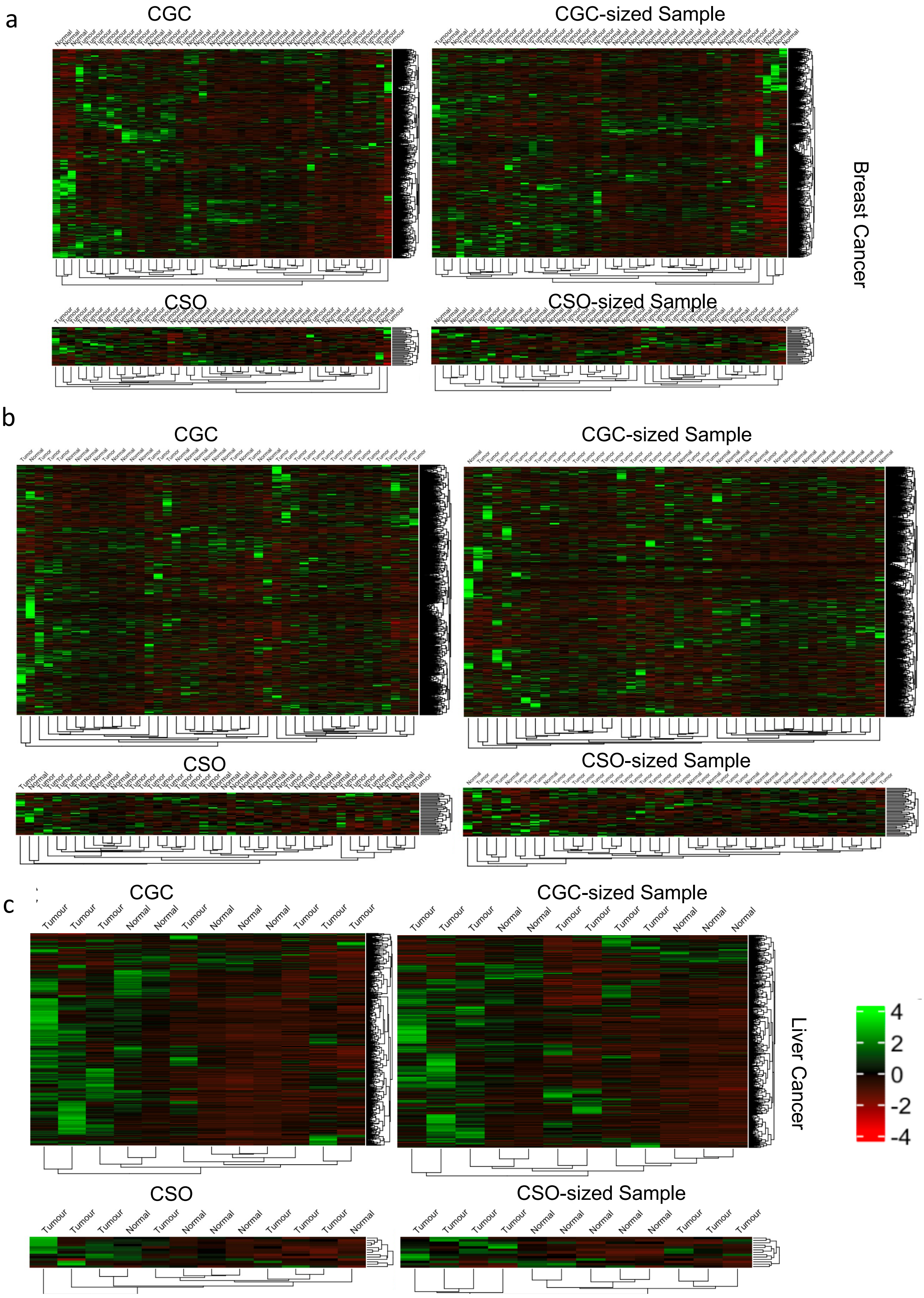
a
CGC
CGC-sized Sample
Breast Cancer
CSO
CSO-sized Sample
b
CGC
CGC-sized Sample
Colorectal Cancer
CSO
CSO-sized Sample
c
CGC
CGC-sized Sample
Liver Cancer
CSO
CSO-sized Sample
4
2
0
-2
-4

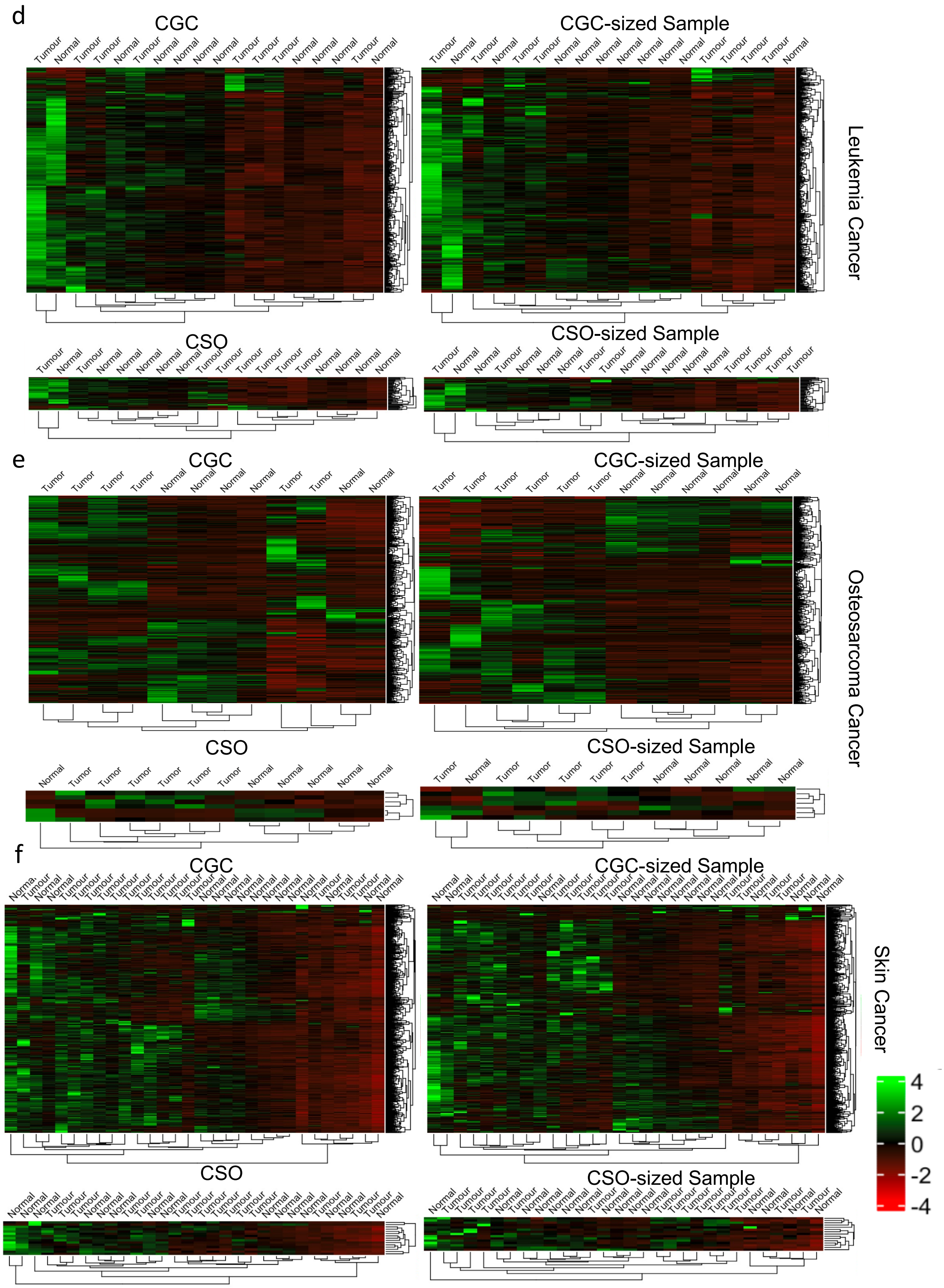

Supplementary Figure 6. Hierarchical clustering of tumor and normal samples for selected cancer types. Hierarchical clustering of cancer and normal samples generated using CGC genes, CSO genes, CGC-sized random sampling of genes, and CSO-sized random sampling of genes in **a)** breast cancer, b) colorectal cancer, **c)** liver cancer, **d)** leukemia, **e)** osteosarcoma, and **f)** skin cancer.

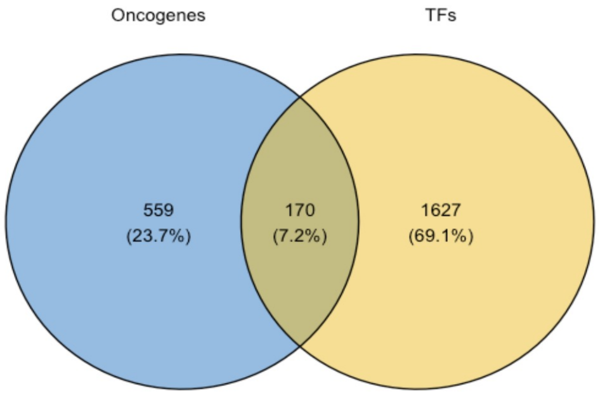

Supplementary Figure 7. Venn Diagram of common genes between CGC and TF. Venn Diagram representing the proportions of CGC genes (blue) that can also be considered transcription factors (yellow).

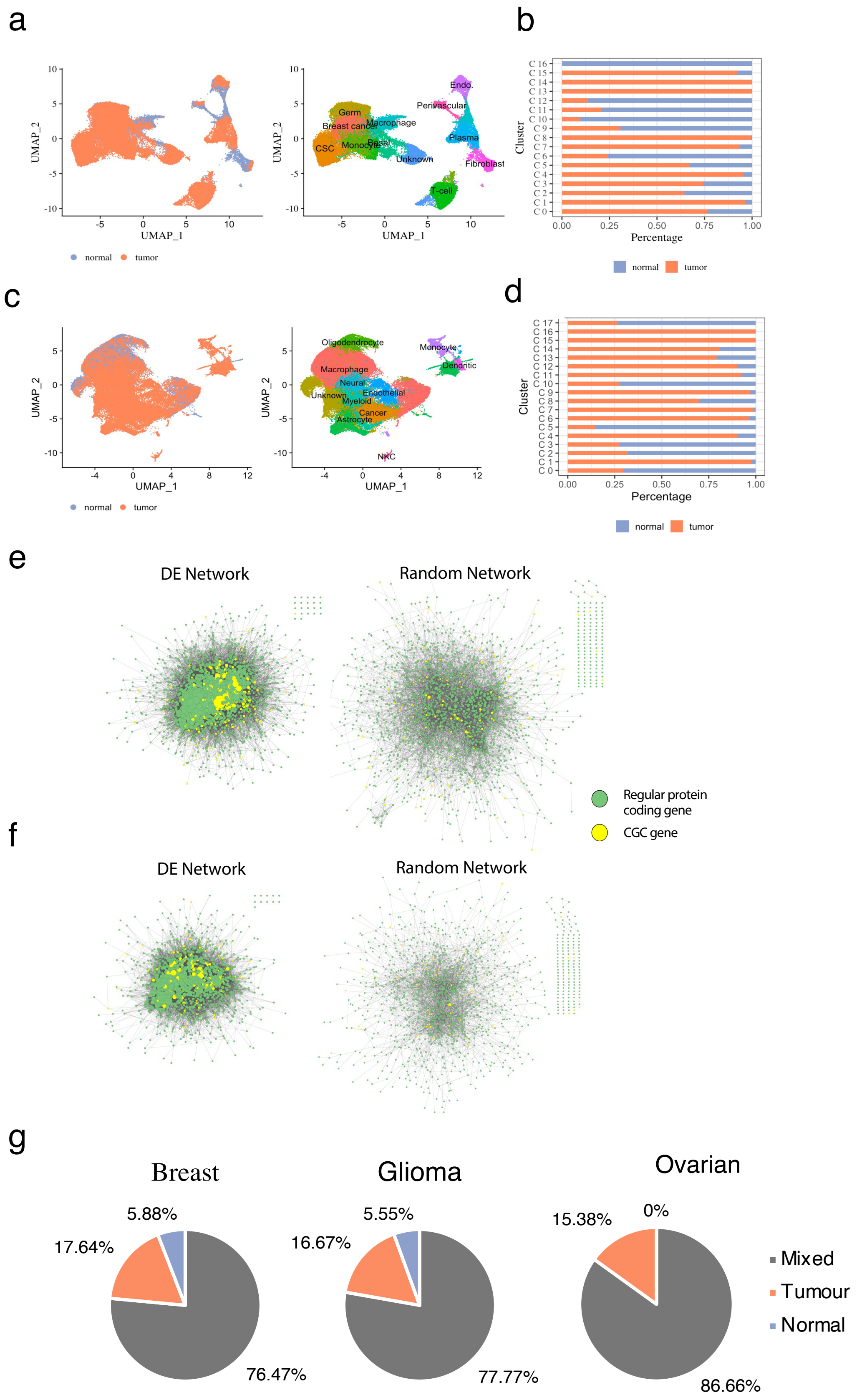
a
UMAP_2
UMAP_1
normal
tumor
Germ
Macrophage
Breast cancer
Basal
CSC
Monocyte
Unknown
Endo.
Perivascular
Plasma
Fibroblast
T-cell
b
Cluster
Percentage
C 16
C 15
C 14
C 13
C 12
C 11
C 10
C 9
C 8
C 7
C 6
C 5
C 4
C 3
C 2
C 1
C 0
0.00
0.25
0.50
0.75
1.00
normal
tumor
c
UMAP_2
UMAP_1
normal
tumor
Oligodendrocyte
Macrophage
Neural
Endothelial
Unknown
Myeloid
Cancer
Astrocyte
NKC
Monocyte
Dendritic
d
Cluster
Percentage
C 17
C 16
C 15
C 14
C 13
C 12
C 11
C 10
C 9
C 8
C 7
C 6
C 5
C 4
C 3
C 2
C 1
C 0
0.00
0.25
0.50
0.75
1.00
normal
tumor
e
DE Network
Random Network
Regular protein coding gene
CGC gene
f
DE Network
Random Network
g
Breast
Glioma
Ovarian
5.88%
17.64%
76.47%
5.55%
16.67%
77.77%
15.38%
0%
86.66%
Mixed
Tumour
Normal

Supplementary Figure 8. scRNA-seq analysis for selected cancer types. a) uMAP dimensional reduction for integrated tumor and normal samples of breast cancer. **b)** Cell condition composition breakdown per cluster in breast cancer. **c)** uMAP dimensional reduction for integrated tumor and normal samples of glioma cancer. **d)** Cell condition composition breakdown per cluster in glioma. **e)** DE networks and random networks generated using Cytoscape with yellow nodes representing CGC genes for breast cancer. **f)** DE networks and random networks generated using Cytoscape with yellow nodes representing CGC genes for breast cancer. **g)** Cluster composition for breast cancer, glioma and ovarian cancer can be categorized into clusters that are predominantly composed of tumor cells (orange), predominantly composed of normal cells (blue), and mixed clusters (grey).

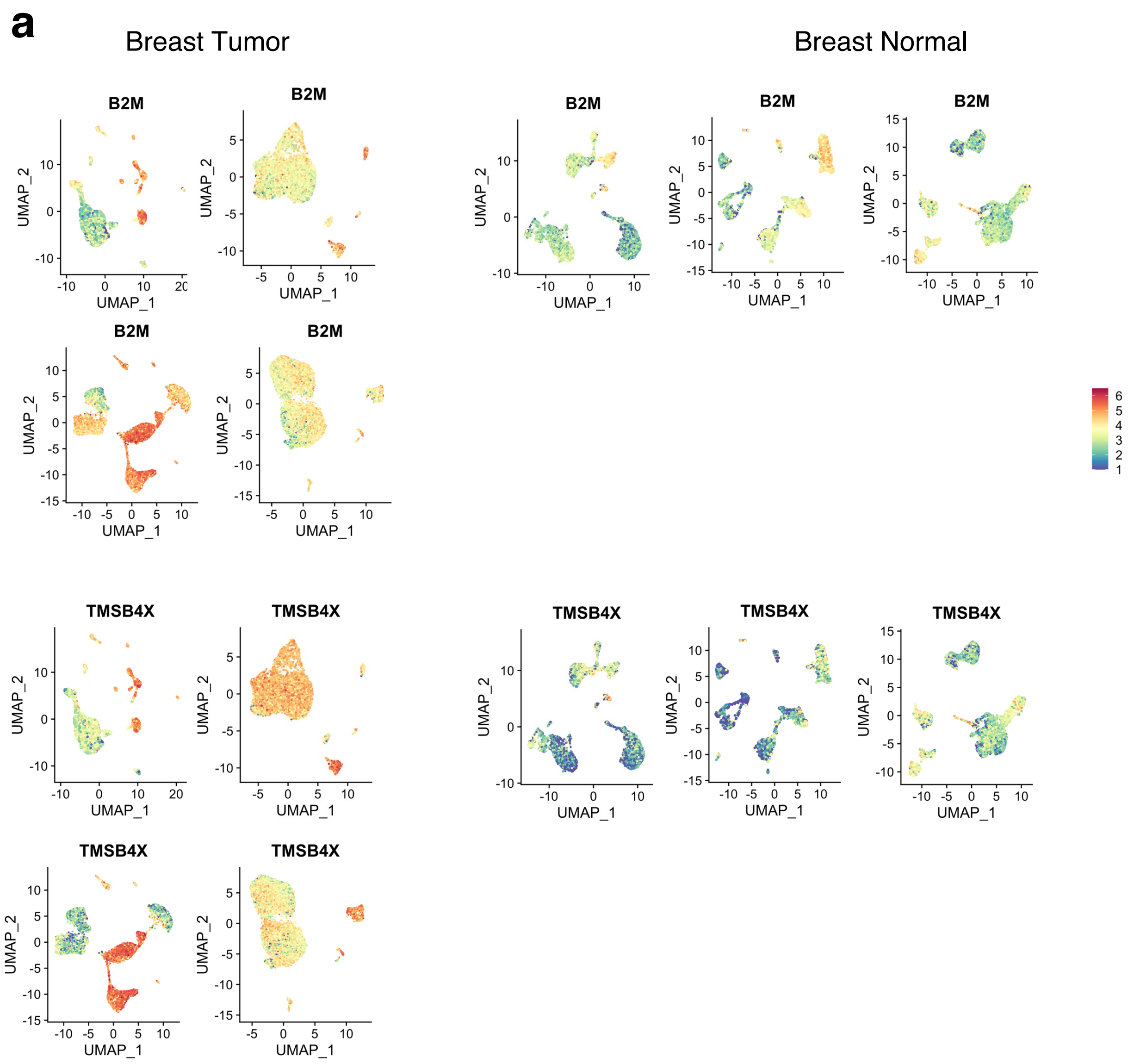
a
Breast Tumor
Breast Normal
B2M
TMSB4X
UMAP_1
UMAP_2

b

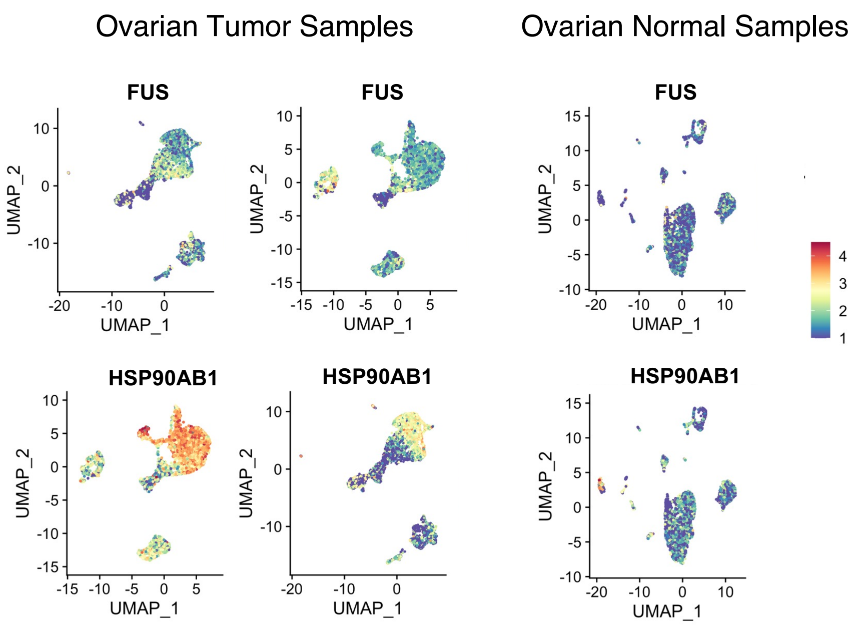

c

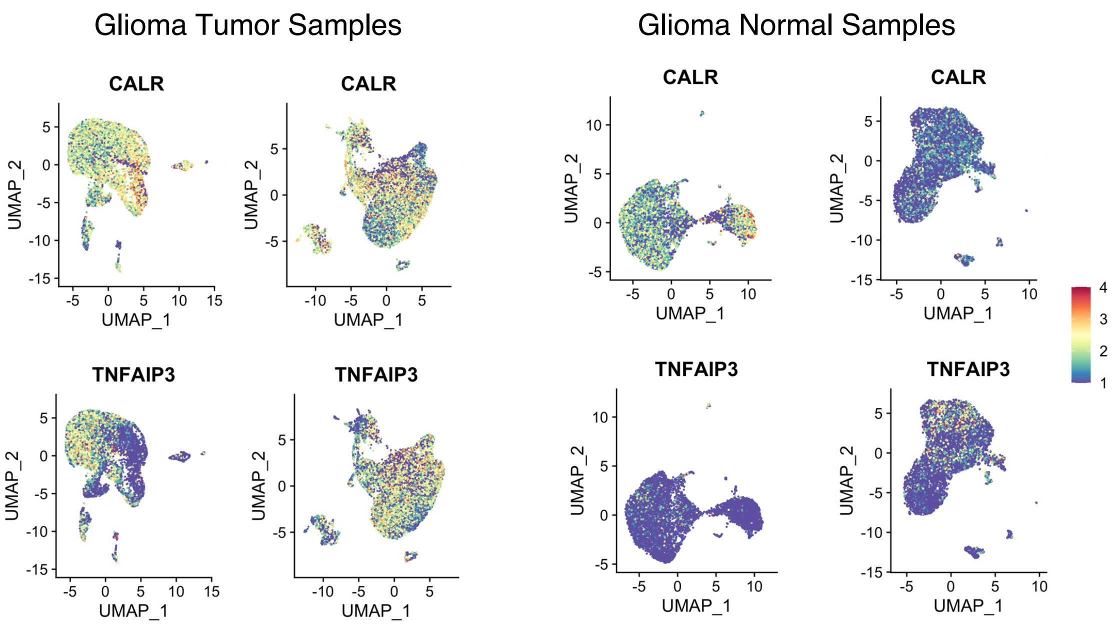

Supplementary Figure 9. Feature Expression plot for top 2 most frequently DE genes show variant expressions between normal and cancer samples. a) breast cancer **b)** ovarian cancer, **c)** glioma cancer. Normal (right panel) and tumor (left panel).

Type	Genes
Breast	BRCA1, CDH1, ARID1B, POLQ, CASP8, MAP2K4, SMARCD1, MAP3K13, KEAP1, PALB2, ESR1, MAP3K1, EP300, SALL4, CTCF, GATA3, CCND1, CDKN1B, ARID1A, PIK3CA, FOXA1, TBX3, BRIP1, BARD1, ETV6, BRCA2, RB1, NTRK3, NCOR1, TP53, ERBB2, AKT1, NOTCH1, BAP1, PBRM1, PPM1D, APOBEC3B, CHEK2
Colorectal	MAP2K4, MLH1, BAX, HIF1A, SALL4, AXIN1, EIF3E, UBR5, SFRP4, FBXW7, QKI, MSH6, PIK3CA, KRAS, APC, MDM2, TP53, SMAD4, AKT1, PIK3R1, RSPO3, TCF7L2, PTPRK, WDCP, TGFBR2, RAD21, B2M, SMAD3, CTNNB1, AXIN2, MAP2K1, SMAD2, POLE, TBL1XR1, GRIN2A, BCL9L, SRC, CUX1
Leukemia	APC, ARID1B, ARID2, AXIN1, AXIN2, CASP8, CTNNB1, DNAJB1, HNF1A, IL6ST, PRKACA, SMAD2, TERT
Liver	LASP1, CREBBP, GAS7, CBFB, KDM5A, PICALM, KAT6A, MECOM, FUS, JAK2, EP300, TRIP11, GATA1, CBL, NUP98, SH2B3, CBLB, RPL22, KMT2A, CSF3R, DNMT3A, DEK, NUP214, KRAS, ABI1, KNL1, LEF1, ETV6, NCOA2, ABL2, ARNT, FANCD2, LPP, ARHGAP26, NSD3, ERG, TAL1, RBM15, RPN1, RAD21, PSIP1, DDX10, PER1, PTPN11, NPM1, BCOR, BCR, MKL1, ARHGEF12, TOP1, CUX1
Ovarian	PIK3R1, BRAF, LRP1B, STK11, ERBB2, FES, AKT2, BRCA1, PTK6, ROS1, PPP2R1A, MSH6, FOXL2, CCNE1, CDK12, PMS2, ARID1B, ARID1A, MLH1, AKT1, RNF43, BARD1, BRCA2, CTNNB1, MAPK1, MSH2, ATR, GOPC, PPM1D
Osteosarcoma	COL1A1, CDH11, EXT2, RECQL4, WRN, CNBP, EXT1
Skin	FAS, ERBB3, SMO, LATS1, DDB2, ERCC5, TBX3, XPA, NOTCH1, XPC, RECQL4, ERCC3, BAP1, STAT5B, POLE

Supplementary Table 1. The subset of CGC genes that are specific to each cancer type.

Dataset	Number of samples	Number of genes/proteins
GSE183947 (breast)	44 normal and 44 tumor	16 980
GSE165255 (Colorectal cancer)	30 normal and 42 tumor samples	16 623
GSE112705 (Hepatocellular carcinoma)	5 normal and 7 tumor samples	13 816
GSE138702 (Acute myeloid leukemia)	10 normal and 8 tumor samples	12 181
GSE190688 (High grade serous ovarian cancer)	5 normal and 6 tumor samples	18 371
GSE126209 (Osteosarcoma)	6 normal and 6 tumor samples	14 966
GSE184616 (Oral squamous cell carcinoma)	15 normal and 15 tumor samples	11 528
PDC00198 (Hepatocellular Carcinoma)	165 normal and 165 tumor samples	7 577
PDC00010 (Ovarian Cancer)	22 normal samples and 85 tumor samples	6218

Supplementary Table 2. Number of samples and genes used in analysis for all cancer types.

Cluster	Type	Genes
0	Basal cells	WFCD2, CLDN3, LCN2, SLPI, CRABP2
1	T-cells	CCL5, CCL4, GZMA, NKG7, GZMB
2	Unknown	KCNQ1OT1, NEAT1, VMP1, VEGFA, MUC16
3	T-cells	LTB, IL7R, TXNIP, CD3D, I L32
4	Dendritic Cells	HLA-DRA, SPP1, HLA-DRB1, CD74, HLA-DPA1
5	B-cells	IGHA1, MUC1, IGHG1, VMP1, MDK
6	Dendrocytes	MTRNR2L8, APOD, KRT14
7	Natural Killer Cells (NKC)	KLRD1,NKG7,KLRC1,CCL5,GNLY
8	Germ cells	CENPF,ASPM,PTTG1,UBE2C,HMGB2
9	Unknown	XIST(lncRNA), IFI44L, GPRIN3, TTN, FYB1
10	Ciliated cells	TPPP3,CAPS,TUBB4B,CFAP126,TSPAN1
11	B-cell/plasma cells	IGHG1, IGHM, IGHA1, JCHAIN, IGHG3
12	Epithelial cells	LGALS4, AGR2, KRT19, CEACAM5, SPINK1
13	Proliferative cells/ Cancer Stem Cells (CSC)	STMN1, MKI67, TUBA1B, ASPM, HMGB2
14	Fibroblasts	COL1A1, COL1A2, COL3A1, DCN, SPARC

Supplementary Table 3. Ovarian Cancer Cluster Markers

Cluster	Type:	Genes
0	Epithelial/ breast cancer cells	KRT19, MUCL1, KRT7, AZGP1, KRT18
1	CSC	CXCL14, CALML5, MUCL1, CD24, CISD3
2	Germ Cells	HIST1H4C, BIRC5, PCLAF, CKS1B, UBE2C
3	Monocytes	S100A9, S100A8, RARRES1, KRT19, FXYD3
4	T-cell	CCL5, IL32, RGS1, TRAC, LTB
5	Basal Cells	MYLK, KRT17, KRT14, ACTA2, FBXO32
6	Macrophage	APOE, LYZ, HLA-DRA, APOC1, HLA-DPA1
7	Plasma Cell	MZB1, FKBP11, CD79A, ITM2C, HERPUD1
8	Unknown	IER2, HSPA6, JUN, JUNB, EGR1
9	Endothelial Cells	IGFBP7, A2M, CD93, SPARCL1, IGFBP3
10	Fibroblast	COL1A2, COL3A1, COL1A1, LUM, DCN
11	Macrophage	SPP1, FTL, APOC1, MT1G, CCL2
12	Perivascular/ fibroblast	IGFBP7, RGS5, COL18A1, PDGFRB, SPARC
13	Unknown	PLA2G16, SERHL2, ADIRF, SCGB2A2, FABP7
14	T-cell	HMGB2, STMN1, RGS1, HIST1H4C, TUBA1B
15	Monocyte	S100A8, C1orf56, S100A9, FABP7, MUCL1
16	Endothelial/ Unknown	FABP4, GNG11, FABP5, HSPB1, TFPI

Supplementary Table 4. Breast Cancer Cluster Markers

Cluster	Type	Genes
0	Macrophage	CCL4L2, CCL4, CCL3L1, CCL2, CCL3
1	Myeloid/ Cancer cell	IGLC2, CXCL2, CXCL3, LYZ, EREG
2	Macrophage	SELENOP, SLC40A1, RNASE1, F13A1, MRC1
3	Macrophage	IGKC, TUBA1B, SPP1, HAMP, FTL
4	Unknown	TNF, JUN, AC245014.3, C12orf57, AC007952.4
5	Oligodendrocyte	CXCL5, TIMP1, CCL20, CXCL3, VCAN
6	Astrocyte	HSPA6, HSPA1A, HSPA1B, DNAJB1, HSP90AA1
7	Myeloid/ microglial	LPL, GPNMB, GLDN, CPM, APOC1
8	Unknown/ neural cells	STMN1, MKI67, TOP2A, CENPF, TUBB
9	Endothelial cells	CLDN5, ANGPT2, SPARC, IGFBP7, ITM2A
10	Unknown	ABL2, IFI30, KDM6B, KCNQ1OT1, SOD2
11	Astrocyte/ endothelial	IGFBP7, MGP, CALD1, SPARCL1, COL4A1
12	Monocyte	S100A9, S100A8, LYZ, S100A12, FCN1
13	Dendritic cell	IGKC, IGLC2, AREG, CCL17, FCER1A
14	NKC	GNLY, IL32, NKG7, CTSW, IL7R
15	Endothelial	COL4A1, SPARCL1, TAGLN, IGFBP7, COL4A2
16	Monocyte	IFITM2, PHACTR1, AQP9, S100A8, S100A9
17	Monocyte	MT2A, MT1E, CTGF, CLDN5, MT1X

Supplementary Table 5. Glioma Cluster Markers
